# Free Circulation of Information and Online Intermediaries – Replacing One “Value Gap” with Another

**DOI:** 10.1007/s40319-020-00982-3

**Published:** 2020-10-13

**Authors:** Valentina Moscon

**Affiliations:** grid.469479.1 0000 0001 1955 4116 Ph.D.; Senior Research Fellow at the Max Planck Institute for Innovation and Competition, Munich, Germany

**Keywords:** Online intermediaries, EU e-Commerce Directive, EU Digital Services Act, Freedom of expression, Safe harbors, Platform self-regulation

## Abstract

The role of online intermediaries in allowing third parties to perform legal as well as illegal activities and the growing economic power of such intermediaries are profoundly challenging the legal framework established 20 years ago with the European Union e-Commerce Directive. European courts first, and legislatures more recently, have taken a position regarding the need for further regulation of online intermediaries. The new liability rules for copyright infringement are just an example of a more general tendency to charge intermediaries with responsibility. This tendency goes beyond the realm of intellectual property and includes consumer law, antitrust and competition law. The forthcoming EU Digital Services Act aims to revise the regulation of online intermediaries by means of new rules framing the responsibilities of digital services and online platforms’ market behaviour.

In this context there is no doubt that the current scenario requires modern rules. However, it is licit to ask whether and how European institutions are considering the collateral effects of the above-mentioned tendency. It is also not clear how the basic principles underpinning the e-Commerce Directive – and, in particular, freedom of expression – will be assured in a new legal framework where online intermediaries may not enjoy the safe harbours originally laid down in the e-Commerce Directive.

Precisely 20 years ago in the European Union, the e-Commerce Directive (ECD)^1^ introduced a conditional liability regime that has so far shielded online intermediaries – or, more accurately “information society service providers” performing mere conduit, caching and hosting – from liability for the unlawful conduct of third parties acting online through their services. For the last two decades, the “safe harbour” regime set forth by the ECD has been a foundational cornerstone for the free circulation of information and the expansion of digital markets. However, over the years the scenario has changed significantly. The rapid development of online intermediaries has allowed new opportunities for information to be freely and massively shared online and made e-commerce a new standard of doing business. At the same time, new issues have arisen. The online dissemination of illegal content is only one of the problems deriving from large use of services offered by online intermediaries. According to the European Commission,^2^ online sales of counterfeit or dangerous products, as well as of other illegally traded goods, are constantly increasing. In addition, the biggest share of the value stemming from digital markets appears to be captured by just a few online intermediaries, which take advantage of their role as gatekeepers and benefit from strong network effects.

The combination of these phenomena – the pivotal role of online intermediaries in allowing third parties to perform legal as well as illegal activities and the growing economic power of such intermediaries – poses a profound challenge to the legal framework established some 20 years ago. European courts initially, and legislatures more recently, have taken a position regarding the need for the further regulation of online intermediaries.

To mention only the most evident patterns, EU jurisprudence has moved along two parallel lines, on the one hand, narrowing the scope of the safe harbours protecting intermediaries from liability for hosting illicit third-party content,^3^ on the other hand, progressively increasing the use of injunctions against online intermediaries.

First, especially in the field of intellectual property rights (IPRs), EU case law has evolved towards depriving internet service providers of their liability privilege by questioning and curbing the concept of a “passive” or “neutral” provider. According to the Court of Justice of the European Union (CJEU),^4^ intermediaries can benefit from the ECD’s safe harbour privileges when they take a mediating, passive or neutral position only. Any further actions involving optimizing the presentation of user-generated content or promoting this content would qualify intermediaries as “active”, thus falling outside the safe harbour of Art. 14 ECD.

The distinction between active and passive providers was also embraced by the European Court of Human Rights (ECtHR), which in *Delfi* found as compatible with the European Convention on Human Rights a decision against an electronic newspaper held liable for the defamatory comments posted by readers in the newspaper’s forum.^5^ The ECtHR qualified as “active” the role of the publisher and even evaluated the word-based filter adopted by the publisher as insufficient for preventing harm being caused to third persons.

At the Member States’ level, the scenario is more nuanced, but it is fair to state that many national courts are following the same approach.^6^ In Italy, for instance, the active or passive role of online intermediaries proved to be relevant not only in the field of IPRs^7^ but even with reference to unfair commercial practices.^8^ In the 2017 *Viagogo* decision, the Italian Competition Authority (Autorità Garante della Concorrenza e del Mercato, AGCM) considered the secondary marketplace for tickets to live events as an “active” intermediary and sanctioned it for deceptive practices performed by its users (for instance, lack of information concerning the original price of tickets, the number of tickets left and the seat location). The decision by the AGCM was eventually reformed by the Italian Supreme Administrative Court (Consiglio di Stato), which held Viagogo as a “passive” hosting provider.^9^ However, it is worth noting that this was not the first case where the AGCM attempted to hold an online intermediary liable for the conduct of its users.^10^

One further relevant development towards enhanced liability of online intermediaries concerns the CJEU’s interpretative effort to determine, under certain conditions, direct (primary) liability rather than merely accessory (secondary) liability of intermediaries in relation to users’ copyright infringements. Over time, the possibility of direct liability for copyright infringement has been envisaged by the CJEU in its case law on the right of communication to the public within Art. 3(1) InfoSoc Directive. In various recent cases, the Court held online intermediaries providing access to users’ content as undertaking acts relevant under Art. 3(1) InfoSoc Directive and therefore potentially liable for copyright infringement.^11^

The second path concerns the use of injunctive reliefs against online intermediaries. As Arts. 12–14 ECD do not affect injunction orders, these have become the most important instrument to contrast illicit content shared through services offered by online intermediaries. Despite the fact that Art. 15 ECD prohibits general monitoring obligations on hosting providers, intermediaries are not relieved from the obligations to comply with injunctions, for instance under Art. 8(3) InfoSoc Directive or Art. 11 Enforcement Directive.^12^ At least in some cases, injunctions have been applied to require providers not only to *take down* actual illicit content, but also to prevent further uploading of the same (or even similar) content (*stay*-*down* obligations).^13^ As a result, online intermediaries are increasingly playing a role in the circulation of information uploaded by third parties.

In line with the above-mentioned judicial approach, among other new legal provisions concerning online intermediaries,^14^ the European legislature recently took a first step toward a real paradigm shift with regard to online intermediaries and their liability for third-party infringements. Article 17 of the Directive on Copyright in the Digital Single Market (DSM Directive)^15^ enacts a direct liability system for online content-sharing services providers (OCSSPs) for copyright infringements committed by their users. Article 17 states that OCSSPs perform an act of communication to the public when they give public access to copyright-protected works uploaded by their users and are liable for such an act unless they fulfil the duty of care as stated in Art. 17(4). Therefore, with specific regard to copyright infringement, Art. 17 derogates from the safe harbour regime of the ECD and opens the way to increased accountability and enhanced liability of online intermediaries.

In the policy and doctrinal debate, it has been discussed – among other relevant issues concerning Art. 17 – whether the liability regime introduced by Art. 17 DSM Directive will solve the alleged mismatch between the value that online content-sharing platforms extract from creative content and the revenue returned to the copyright holders (the so-called "value-gap"). In any case, it is clear that the new liability rules for copyright infringement are just an example of a more general tendency, which goes beyond the copyright realm and includes, to mention one more field, antitrust and competition law. Both in the EU and the US, lately – at least before COVID-19 – the main concern of competition authorities seemed to be reducing the economic and political power of large online platforms (especially those labelled as GAFA).^16^

Finally, in February 2020 the EU Commission announced a Digital Services Act Package.^17^ The package is intended to modernise the current legal framework for digital services by means of two main pillars: first, new rules framing the responsibilities of digital services to address the risks faced by their users and to protect their rights; and second, *ex ante* rules covering large online platforms acting as gatekeepers, which should ensure that those platforms behave fairly and can be challenged by new entrants and existing competitors. The proposals for EU regulations should be ready by the end of 2020.^18^

These judicial and legislative initiatives are already triggering reactions from online intermediaries, both through lobbying at the level of policy debate and – what is perhaps more critical – through self-regulation. In May 2020, for instance, Facebook announced the creation and empowerment of a new “Oversight Board” to exercise independent judgment over some of the most difficult and significant content decisions.^19^

In these circumstances it is licit to ask whether and how European institutions are considering the collateral effects of the emerging trend “against” online intermediaries. It is beyond dispute that the current scenario is very far from that of 20 and more years ago. As correctly pointed out by the European Commission with respect to the preliminary works for the new Digital Services Act Package, “The European Single Market requires a modern legal framework to ensure the safety of users online and to allow innovative digital businesses to grow, while respecting the basic principles underpinning the current legal framework of the e-Commerce Directive”. At the moment, however, it is not clear how these principles – and, in particular, freedom of expression – will be practically assured in a new legal framework where online intermediaries may not enjoy the safe harbors originally laid down in the ECD. In other words, there is an urgent need to ask what legal instruments will be adopted in the EU to assure freedom of expression in a digital environment where online intermediaries will be potentially liable for the illicit conducts of third parties using their services.

While the specific interests of some stakeholders will be adequately represented during the legislative process, “The Logic of Collective Action”^20^ teaches us that, presumably, fundamental values like freedom of expression and free circulation of information risk being underestimated in the political debate. And this might be the “new value gap”. The risk is that the new rules will just replace one “value gap” with another. Without overestimating the role and weight of academia in the political arena, it seems reasonable to say that in the near future European scholars (and not only them) should step into the technical debate, by designing and proposing workable solutions for actual protection of these fundamental values in the new scenario.

## Footnotes

1 Directive 2000/31/EC of the European Parliament and of the Council of 8 June 2000 on certain legal aspects of information society services, in particular electronic commerce, in the Internal Market, OJ L 178, 17.7.2000, pp. 1–16.

2 *See* European Commission, Combined Evaluation Roadmap/Inception Impact Assessment, Ares (2020)2,877,647. Available at https://ec.europa.eu/info/law/better-regulation/have-your-say/initiatives/12418-Digital-Services-Act-package-ex-ante-regulatory-instrument-of-very-large-online-platforms-acting-as-gatekeepers.

3 *See* Riordan (2016), p. 379.

4 *L’Oréal SA and Others v. eBay International AG and Others*, C-324/09, ECLI:EU:C:2011:474; *Google France SARL and Google Inc. v. Louis Vuitton Malletier SA* (C-236/08), *Google France SARL v. Viaticum SA and Luteciel SARL* (C-237/08), *Google France SARL v. Centre national de recherche en relations humaines (CNRRH) SARL and Others* (C-238/08), ECLI:EU:C:2010:159. *See* more in Rosati (2019).

5 *Delfi v. Estonia,* App No. 64569/09 (ECtHR, 16 June 2015). In a similar direction, *Magyar Tartalomzolgalatok Egysuelete and Index.hu Zrt v. Hungary,* App No. 22947/13 (ECtHR, 2 February 2016).

6 *See* EUIPO, IPR Enforcement Case-Law Collection, August 2019, available at https://euipo.europa.eu/tunnel-web/secure/webdav/guest/document_library/observatory/documents/reports/2019_IPR_Enforcement_Case_Law_Collection/2019_IPR_Enforcement_Case_Law_Collection_en.pdf.

7 In *RTI* the Supreme Court of Cassation detailed the activities that make a hosting provider “active” and therefore not shielded by Art. 14 ECD. *See* “Internet Hosting Provider Liability for Copyright Infringement – *RTI v. Yahoo Italia*”, IIC 51:389–402 (2020).

8 On this matter, *see* Hilty and Moscon (2020).

9 AGCM, *VIAGOGO – Secondary Market,* PS10610 (It.); Consiglio di Stato, 25 June 2019, Decision No. 4359/2019 (It.).

10 AGCM, *TripAdvisor*, PS9345, paras. 87–9 (It.).

11 *See* among others *GS Media BV v. Sanoma Media Netherlands BV and* *Others,* C-160/15, ECLI:EU:C:2016:644; *Stichting Brein v. Ziggo BV and XS4All Internet BV,* C-610/15, ECLI:EU:C:2017:456. Against this approach, *see* the recent AG Opinion in *Frank Peterson v. Google LLC*, *YouTube LLC, YouTube Inc., Google Germany GmbH,* C-682/18 and *Elsevier Inc. v. Cyando AG,* C-683/18, ECLI:EU:C:2020:586.

12 Directive 2001/29/EC of the European Parliament and of the Council of 22 May 2001 on the harmonisation of certain aspects of copyright and related rights in the information society; Directive 2004/48/EC of the European Parliament and of the Council of 29 April 2004 on the enforcement of intellectual property rights.

13 *L’Oréal SA and Others v. eBay International AG and Others,* C-324/09, ECLI:EU:C:2011:474; *Tommy Hilfiger Licensing LLC and Others v. Delta Center a.s.*, C-494/15, ECLI:EU:C:2016:528. For a general overview on the matter, *see* Husovec (2020), pp. 90–104.

14 Regulation (EU) 2019/1150 of the European Parliament and of the Council of 20 June 2019 on promoting fairness and transparency for business users of online intermediation services; Directive (EU) 2018/1808 of the European Parliament and of the Council of 14 November 2018 amending Directive 2010/13/EU on the coordination of certain provisions laid down by law, regulation or administrative action in Member States concerning the provision of audiovisual media services (Audiovisual Media Services Directive) in view of changing market realities; European Commission, “Commission Recommendation on measures to effectively tackle illegal content online” (1 March 2018) (C(2018) 1177 final).

15 Directive (EU) 2019/790 of the European Parliament and of the Council of 17 April 2019 on copyright and related rights in the Digital Single Market and amending Directives 96/9/EC and 2001/29/EC.

16 *Ex multis*, *see* Khan (2017); Wu (2018).

17 European Commission, Communication: Shaping Europe’s digital future, 19 February 2020, available at https://ec.europa.eu/info/sites/info/files/communication-shaping-europes-digital-future-feb2020_en_4.pdf.

18 *See* https://ec.europa.eu/info/law/better-regulation/have-your-say/initiatives/12418-Digital-Services-Act-package-ex-ante-regulatory-instrument-of-very-large-online-platforms-acting-as-gatekeepers and https://ec.europa.eu/info/law/better-regulation/have-your-say/initiatives/12417-Digital-Services-Act-deepening-the-Internal-Market-and-clarifying-responsibilities-for-digital-services.

19 *See* https://about.fb.com/news/2020/05/welcoming-the-oversight-board/.

20 Olson (1965).
